# Analysis of the Influence of Diabetic Nephropathy in Patients with Diabetic Foot Osteomyelitis

**DOI:** 10.3390/jcm12175557

**Published:** 2023-08-26

**Authors:** Yolanda García-Álvarez, Francisco Javier Álvaro-Afonso, Marta García-Madrid, Aroa Tardáguila-García, Mateo López-Moral, José Luis Lázaro-Martínez

**Affiliations:** Diabetic Foot Unit, Clínica Universitaria de Podología, Facultad de Enfermería, Fisioterapia y Podología, Universidad Complutense de Madrid, Instituto de Investigación Sanitaria del Hospital Clínico San Carlos (IdISSC), 28040 Madrid, Spain; ygarci01@ucm.es (Y.G.-Á.); magarc28@ucm.es (M.G.-M.); aroa.tardaguila@ucm.es (A.T.-G.); matlopez@ucm.es (M.L.-M.); diabetes@ucm.es (J.L.L.-M.)

**Keywords:** diabetic foot, diabetic foot ulcers, diabetic foot infection, diabetic foot osteomyelitis, diabetic nephropathy, conservative surgery

## Abstract

This study analyzed the influence of diabetic nephropathy on the healing prognosis after conservative surgery in diabetic foot osteomyelitis (DFO). A retrospective observational study was carried out between January 2021 and December 2022 and involved 278 outpatients with a diagnosis of DFO at a specialized diabetic foot unit, including 74 (26.62%) patients with DN (group 2) and 204 (73.38%) patients without DN (group 1). There were 266 (95.70%) ulcers on the forefoot, 8 (2.90%) on the midfoot, and 4 (1.45%) on the hindfoot (*p* = 0.992). The healing rates were 85.1% (*n* = 63) for group 2 and 81.3% (*n* = 165) for group 1 (*p =* 0.457). When exploring the influence of DN on the risk of delayed ulcer healing, the results did not show a significant effect [12 (6; 28) weeks among patients with DN vs. 12 (6; 21) weeks among patients without DN; *p* = 0.576]. No significant differences were observed in complications, with one (2.59%) death occurring in group 1 (*p* = 0.296) and three minor amputations being performed in both groups [two (5.13%) amputations in group 1 vs. one amputation (9.09%) in group 2; *p* = 0.217]. Bone cultures were performed for a total of 190 patients (133 in group 1 and 57 in group 2). Of these, 176 positive bone cultures were isolated: 71 positive bone cultures (57.7%) were monomicrobial cultures in group 1, with 30 (56.6%) in group 2. There were 52 (42.3%) that had at least two microorganisms in group 1, and 23 (43.4%) in group 2 (*p* = 0.890). The most frequently prescribed oral antibiotic was amoxicillin/clavulanate (43.89%), followed by levofloxacin (28.4%), and trimethoprim/sulfamethoxazole (14.7%). This study shows that DN does not have a significant influence on the healing prognosis of patients with DFO after conservative surgery.

## 1. Introduction

Diabetic nephropathy (DN) is a chronic and common microvascular complication that occurs in 20–40% of patients with diabetes mellitus (DM) [1,2] and it is the leading cause of ESRD worldwide [3]. It can progress to end-stage renal disease (ESRD), which requires dialysis or kidney transplantation [3]. DN also has a well-recognized relationship with diabetic foot disease and occurs in 39.3% of patients [4,5].

Different levels of decreased renal function impact the foot, cardiovascular system, and survival when a foot ulcer occurs in patients with DM [5]. Patients with DN are prone to peripheral arterial disease (PAD) with a 2.5-fold greater risk [6], as well as neuropathy and infections [7,8]. Diabetic foot osteomyelitis (DFO) is the most common infection associated with DFU and remains a diagnostic and therapeutic challenge for clinicians, especially in patients with severe comorbidities [9] such as DN [10,11,12].

DN is an independent risk factor for the development of ulceration [7] and worse DFU outcomes [4,5], including lower ulcer healing and the highest rates of minor and major amputation [5]. There is a strong association between the stage of DN and DFU or amputation [13]. The greatest association is for patients with the most severe DN or those on dialysis therapy [14]. However, even less severe cases are approximately two times more likely to develop DFU or undergo amputation than those with minimal to no impairment [13,15,16]. Additionally, these patients have a higher risk of mortality, especially due to cardiac events [5] and subsequent postoperative procedures among those with moderate to severe DN [17] and those on dialysis therapy [18,19]. They also have the highest rate of major postoperative complications, such as repeat amputation at the same level or higher [17].

There is a lack of evidence regarding the presence of DN associated with DFO. A recent study has demonstrated the importance of elevated albuminuria, vibration perception, and microcirculation disorders, which have key roles in the recurrence of foot ulcers in patients with DFO and DN [20]. Most studies have tried to relate the influence of DN to DFU, amputation, or death according to the degree of renal impairment based on the estimated glomerular filtration rate (eGFR) [5], creatinine clearance [21], or albuminuria [22,23]. However, the presence of DFO has not been taken into account. It has been demonstrated that patients with DN and DFO have a significantly higher risk of cardiovascular mortality and all-cause mortality than patients with diabetic foot disease alone [5,20], which is probably associated with the high severity of such patients.

To the best of our knowledge, no study in the literature has analyzed the relationship of DN with microbiological profiles and wound characteristics in DFU complicated by osteomyelitis. Therefore, the main objective of this study was to explore the influence of diabetic nephropathy on the healing prognosis after conservative surgery. The secondary objective was to evaluate the local and pathogenic microorganism diversity of patients with DFU complicated by DFO.

## 2. Methods

### 2.1. Study Design and Participants

A retrospective observational study was conducted between January 2021 and December 2022 in a specialized diabetic foot unit. In total, 278 patients diagnosed with DFO with or without DN were included. Patients were eligible if they had DM, aged over 18 years, DFU complicated by DFO treated with surgery, at least grade 3 “O” according to the PEDIS classification [24], and at least grade IIIB/IIID according to the Texas classification [25]. Patients who had received medical treatment for DFO during 6 weeks according to the recommendations of the IWGDF Guide published in 2016 [26] or had a diagnosis of critical ischemia were excluded.

### 2.2. Patient Assessment

All patients underwent neurological and vascular screening. The neurological screening consisted of the evaluation of superficial sensitivity using Semmes–Weinstein monofilament (5.07/10 g monofilament—Novalab Ibérica, Alcalá de Henares, Madrid, Spain) and deep sensitivity using the biotensiometer (Horwell biotensiometer Me.Te.Da. S.r.l., San Benedetto del Tronto, Italy). Patients were considered neuropathic in cases where at least one of the two test results was positive [27].

Vascular screening consisted of the palpation of distal pulses (pedal and posterior tibial), ankle–brachial index (ABI), toe–brachial index (TBI), and transcutaneous oxygen pressure (TcPO_2_). Patients were considered to have peripheral arterial disease (PAD) when pulses were not patent, the ABI was less than 0.9, the TBI was less than 0.7, and TcPO_2_ was less than 30 mmHg [28].

### 2.3. DFO Diagnosis

Clinical diagnosis of DFO was performed using the probe-to-bone (PTB) test and plain X-ray, a diagnostic combination that shows high sensitivity and specificity values [29,30]. Both tests had to be positive to consider the presence of DFO. The PTB test was considered positive when the bone was palpated through the DFU with sterile mosquito forceps. The X-ray result was considered positive when the affected bone showed changes compatible with DFO, such as cortical disruption and periosteal elevation or sequestrum, among other radiological signs that have been described in the literature [24].

### 2.4. DFO Management

All patients were managed surgically by a single surgeon with more than 20 years of experience in conservative diabetic foot surgery (J.L.M.). Conservative surgery for the management of DFO is described as the removal of bone and necrotic soft tissue without amputating any part of the foot [31,32]. In addition to the surgical intervention, all patients were prescribed culture-guided post-surgical antibiotic treatment [33]. Additionally, following protocol in our Unit, antibiotics are maintained for 7–10 days after a conservative surgery, as recommended by international guidelines for the management of diabetic foot infection. In cases where the culture was negative, empirical antibiotics were prescribed [34].

### 2.5. Bone Samples

During surgery, bone samples were obtained for microbiological culture and histopathological analysis [24]. The samples were obtained using aseptic technique, ensuring disinfection and avoiding contamination by washing with povidone–iodine and rinsing with saline solution [33]. Samples were immediately transported to the microbiological laboratory for analysis. For microbiological analysis, each of the bone-segment samples was weighed and mechanically homogenized for 5 min in 1 mL sterile phosphate-buffered saline, pH = 7.4 (PBS; Sigma Aldrich, St. Louis, MI, USA). Homogenates were transferred into a new tube. Undiluted samples and samples diluted in PBS (1:10–1:100) were plated (100 µL) onto Columbia (BD; Sparks) and MacConkey agars (BD) using a spiral platter workstation (Don Whitley Scientific, West Yorkshire, UK). Plates were incubated at 35 °C for 24 h. All microorganisms were identified by conventional methods (Gram staining followed by biochemical techniques based on API-bioMérieux or BBl Crystal ID-BD). The specific enumeration of organisms was directly performed on the Columbia agar plates using a stereomicroscope (SteREO Discovery V.8, Carl Zeiss Microimaging, Jena, Germany), after comparing the morphology of grown colonies with those observed in the pure cultures from qualitative analysis [35].

### 2.6. DN Assessment

DN or kidney disease was confirmed to be present in cases in which the values obtained by blood tests at the time of diagnosis of DFO showed a glomerular filtration rate lower than 60 mL/min/1.73 m^3^ [36] and creatinine values higher than 1.3 mg/dL [37]. No further renal function data were obtained, including urinalysis or imaging tests.

### 2.7. Statistical Analysis

Statistical analysis was carried out using SPSS^®^ statistical software version 28.0 for iOS (SPSS, Inc. Chicago, IL, USA). Means and standard deviations were calculated for quantitative variables, and frequencies and percentages were calculated for qualitative variables. In the case of quantitative variables, normality was assessed using the Kolmogorov–Smirnov test, Student’s *t*-test was used for normally distributed variables, and the Mann–Whitney U test was used for non-normally distributed variables. The chi-squared test was used to compare qualitative variables. The Kaplan–Meier method was used to assess survival time to healing, and the log-rank test was used to compare survival time between patients with and without DN. Values of *p* < 0.05 were considered statistically significant with a 95% confidence interval.

### 2.8. Ethics

This study has been approved by the local Ethics Committee (code 23/092-E_TFM; Ethics Committee of the Hospital Clínico San Carlos, Madrid, Spain). Due to the retrospective nature of the study, the collection of informed consent from patients was not required, and the authors declare that the study was conducted in accordance with the ethical standards of the Declaration of Helsinki [38].

## 3. Results

### 3.1. Clinical Characteristics of Patients

In total, 278 DFO patients with complete data were included in the analysis, including 74 (26.62%) patients with DN and 204 (73.38%) patients without DN. The study population was predominantly male (77.7%), and the mean age was 63 years (range, 58–73 years). There were 244 patients (88.1%) who had type 2 diabetes, whereas the remainder had type 1 diabetes. The mean duration of diabetes was 15.00 (9.25; 24.75) years, and the mean hemoglobin A1c value was 7.5% (range, 6.5–8.55) (58 (48–69) mmol/mol) [39]. Of the 74 patients with DN, 52 (70.27%) patients received dialysis or had ESRD. Differences in PAD, infection severity, and diabetic retinopathy were statistically significant between the two groups (*p* = 0.020, *p* = 0.020, and *p* = 0.001, respectively). There were no significant differences in age, sex, duration and type of diabetes, smoking, alcoholism, hypertension, hypercholesterolemia, CHD, neuropathy, HbA1c, distal pulses, ABI, duration of wound, and infection severity between groups. The patient characteristics are summarized in Table 1.

### 3.2. Analysis of Ulcer Locations in Patients with DFO and DN

In both groups, the most prevalent ulcer location was the forefoot, and there were no significant differences between groups. There were 266 (95.70%) ulcers on the forefoot, 8 (2.90%) on the midfoot, and 4 (1.45%) on the hindfoot (*p* = 0.992). Table 2 shows the frequency of locations of foot ulcers in the study population.

### 3.3. Relationship of Diabetic Nephropathy with Ulcer Healing Times and Rates

**Figure 1:**
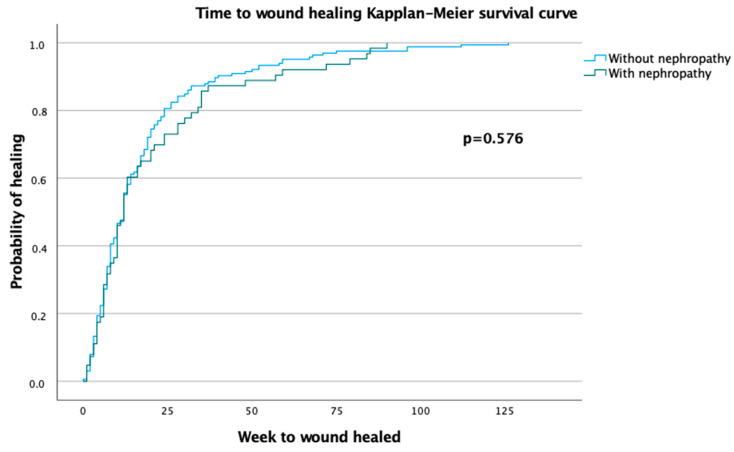
Kaplan–Meier survival curve of time to wound healing.

The durations of ulcers in patients with DN versus patients without DN were 15 (3.75, 26.50) and 8 (3, 24.25) weeks, respectively (*p =* 0.471). The healing rates were 85.1% (*n* = 63) for patients with DN and 81.3% (*n* = 165) for patients without DN (*p =* 0.457). When exploring the influence of DN on the risk of delayed ulcer healing, the results did not show a significant effect (12 (6; 28) weeks among patients with DN vs. 12 (6; 21) weeks among patients without DN; *p* = 0.576). The Kaplan–Meier estimates are presented in Figure 1.

In the group of patients who healed with DN, 45 (60.81%) had hemodialysis, and their mean healing time was 12 (6, 33) weeks. For patients with DN who were not on dialysis, the mean healing time was 5.5 (11.0, 20.2) weeks (*p =* 0.437). Healing rates were 81.8% (*n* = 18) among patients with DN and without hemodialysis and 86.5% (*n* = 45) among patients with DN and hemodialysis (*p =* 0.602).

When we analyzed the group of hemodialysis patients (52, 18.71%) vs. the rest of the patients (226, 81.30%), significant differences were not observed in healing rate among those with DFU and dialysis (45, 86.5%) compared with those without dialysis (184, 81.42%) (*p* = 0.375). Similarly, significant differences were not observed in the mean healing time among those with DFU and hemodialysis (12 (6, 20)) compared with those with DFU without hemodialysis (12 (6, 33)) weeks; *p =* 0.347). In the group of patients who did not heal (*n* = 50), 39 (78%) did not have DN (group 1), and 11 (22%) had DN (group 2), 7 of these patients were on dialysis.

No significant differences were observed in complications, with one (2.59%) death occurring in group 1 (*p* = 0.296) and three minor amputations being performed in both groups (two (5.13%) amputations in group 1 vs. one amputation (9.09%) in group 2; *p* = 0.217).

### 3.4. Bacterial Diversity Isolated among Patients with DFO Stratified by the Presence of DN

Bone cultures were performed for a total of 190 patients (133 among patients without DN and 57 patients with DN). Of these, 176 positive bone cultures were isolated (92.6% of all bone cultures, 92.5% among patients without DN (*n* = 123), and 93% among patients with DN, *p* = 0.904)). There were 71 positive bone cultures (57.7%) that were monomicrobial cultures in group 1 and 30 (56.6%) in group 2. There were 52 (42.3%) that had at least two microorganisms in group 1 and 23 (43.4%) in group 2 (*p* = 0.890). Table 3 shows the frequency of the isolated microorganisms. The most frequently prescribed oral antibiotic was amoxicillin/clavulanate (43.89%), followed by levofloxacin (28.4%), trimethoprim/sulfamethoxazole (14.7%), clindamycin (13.7%), linezolid (5,4%), cloxacillin (3.9%), and erythromycin (3.6%).

## 4. Discussion

Conservative surgical treatment of diabetic foot osteomyelitis has been demonstrated to be safe and effective [40]. In this study, we observed that DN does not have a significant influence on the healing prognosis of patients with DFO after conservative surgery. We observed that healing rates were 85.1% (*n* = 63) among patients with DN compared with 81.3% (*n* = 165) among patients without DN (*p =* 0.457). When exploring the influence of DN on the risk of delayed ulcer healing, the results did not show a significant effect on the association of DN and longer healing times or probability of healing (12 (6; 28) weeks among patients with DN vs. 12 (6; 21) weeks among patients without DN; *p* = 0.576; see Figure 1).

When stratifying patients with DN according to whether they received dialysis treatment, we did not find statistically significant differences in healing rates or time to healing. Healing rates were 81.8% (*n* = 18) among patients with DN without hemodialysis compared with 86.5% (*n* = 45) among patients with DN and hemodialysis (*p* = 0.602). The mean healing time was 12 (6,33) weeks among patients on dialysis versus 5.5 (11.0, 20.2) weeks among patients with DN and no dialysis (*p* = 0.437). These results suggest that when treatment is based on early and conservative surgical debridement, DN is not associated with worse prognosis among patients with DFO.

Meloni et al. [41,42] observed that dialyzed patients had a higher risk of non-healing and major amputation than those with preserved renal function. They also found that patients with comorbid kidney disease and cardiovascular disease have a poor prognosis. Along the same line, Caruso et al. [43] concluded that wound healing rates are reduced in cases of diabetes with kidney disease. Li et al. [20] showed that microcirculation disorders play an important role in the recurrence of ulcers among patients with DFO and DN. However, no other study has assessed the evolution of DFO after treatment with conservative surgery; therefore, the previous data cannot be compared with those obtained in this study.

Regarding clinical characteristics, statistically significant differences were found among patients without and with DN in the duration of diabetes (15 (7.75; 22.50) vs. 19 (10.75; 25) years, respectively, *p* = 0.046), PAD (40.7% vs. 56.8%, respectively, *p* = 0.020), infection severity (Texas IIID 40.7% vs. 56.8%, respectively, *p* = 0.020), and diabetic retinopathy (27% vs. 47.3%, respectively, *p* = 0.001). These differences between groups were expected because DN develops in approximately 40% of patients with diabetes in 7 to 10 years after the diagnosis of type 2 diabetes mellitus [44]. Patients with DN are prone to PAD with a 2.5-fold higher risk [45]. Garimella et al. [46] observed that chronic kidney disease is commonly complicated by PAD, which impairs the wound-healing process in DFU. However, evidence on the presence of DN associated with DFO is lacking.

Zhang et al. [20] recently showed the importance of elevated albuminuria, vibration perception, and microcirculation disorders in the recurrence of foot ulcers among patients with DFO and DN. DN and diabetic retinopathy are microvascular complications of diabetes [47]. The coincidence of retina and kidney pathologies in diabetic patients is well recognized, and certain studies have proposed a definition of “renal-retinal syndrome” [47].

No significant differences were found between patients without and with DN in wound duration (8 (3; 24.25) vs. 12 (3.75; 26.50), *p* = 0.471) or ulcer location, of which the most prevalent was the forefoot in both groups. We did not find significant differences among groups in the number of monomicrobial and polymicrobial infections. There were 71 positive bone cultures (57.7%) that were monomicrobial cultures in group 1 and 30 (56.6%) in group 2. There were 52 (42.3%) that had formed with at least two microorganisms in group 1 and 23 (43.4%) in group 2 (*p* = 0.890). Therefore, the same behavior was observed among patients with and without kidney disease. Similarly, we observed no differences in bacterial diversity between groups (see Table 3)**.**

The present data are similar to previous studies, where approximately 62.7% of cases had monomicrobial bone cultures, and 37.3% were formed with at least two microorganisms [48]. Significant differences were observed for only methicillin-resistant *Staphylococcus aureus* (MRSA) (*p* = 0.017). MRSA has become increasingly common in diabetic foot infections (DFIs) [45,49]. The risk factors for MRSA in DFIs include antibiotic use in the 6 months prior to hospitalization, inappropriate use of antibiotics, previous hospitalization, long duration of the ulcer, osteomyelitis, hypoproteinemia, larger ulcers, and the nasal transport of MRSA [50,51,52]. In patients with kidney disease, there is a higher prevalence of hospital stays that could influence the appearance of more severe pathogens; however, in this study, it was observed that if correct surgery is performed for osteomyelitis, there are no differences in the prognosis for healing. These results are consistent with previous studies.

In this regard, Álvaro et al. [53] compared patients with DFI with MRSA and MSSA treated by surgical debridement. They did not find significant differences among groups in the number of surgical procedures to resolve infection (15 (41.7%) vs. 13 (33.3%), *p* = 0.456) or in healing times among patients treated by a surgical procedure (10.5 weeks (6.7;16.5) vs. 6.1 weeks (3;8.7), *p* = 0.068). This suggests that when treatment is early and based on surgical debridement, MRSA infections are not associated with a worse prognosis. However, the role of MRSA in DFIs remains unclear and complicated, due to the meaning of MRSA and its influence on clinical evolution.

All of our patients received standard care for their wounds, which consisted of debridement, moist wound dressings for wound management, and proper off-loading. Patients received empirical antibiotics chosen according to IDSA guidelines [34]. The antibiotic therapy was adjusted to target isolated bacteria based on tissue-culture results. In the case of patients with DN, the prescription was adjusted according to nephrology results. The most frequently prescribed oral antibiotic was amoxicillin/clavulanate (43.89%), followed by levofloxacin (28.4%), trimethoprim/sulfamethoxazole (14.7%), clindamycin (13.7%), linezolid (5,4%), cloxacillin (3.9%), and erythromycin (3.6%). The most frequently prescribed oral antibiotics were similar to those observed in previous studies [48].

There were several limitations in our study. First, the study cohort was a Spanish population of outpatients with DFO from a single center who were treated surgically. Thus, the results may not be generalizable to populations with different demographic characteristics or hospitalized patients, and selection bias is inevitable. Second, we used a retrospective cohort design that included data collection from electronic medical records created for patient care, not for research, and it was not possible to obtain urine samples for other diagnostic tests, such as hematuria or proteinuria, so the results may suggest a diagnostic bias. Nevertheless, the baseline characteristics were similar between groups.

Third, we did not categorize the different degrees of kidney disease. We only stratified patients according to the presence of DN except for patients with dialysis or ESRD, which were recorded and analyzed separately.

## 5. Conclusions

To the best of our knowledge, this is the first study comparing patients with and without kidney disease to examine whether ND is associated with a poorer prognosis for cure among patients with DFO after conservative surgery. We observed that the DN does not have a significant influence in this sense. However, patients with ND were found to be more likely to have MRSA diabetic foot infections, although no significant difference was observed between groups in median healing time after conservative surgical treatment. Future studies are needed to analyze the influence of ND on the healing prognosis in all types of DFU.

## Figures and Tables

**Table 1:** Clinical characteristics.

Clinical Data	Without DN	With DN	*p* Value
	(Group 1)	(Group 2)	
	(*n* = 204)	(*n* = 74)	
Age, years	64.48 ± 11.76	63.42 ± 11.57	0.602
Sex, *n* (%)			
Female	50 (24.5%)	12 (16.2%)	0.142
Male	154 (75.5%)	62 (83.3%)	
Diabetes duration (years)	15 (7.75; 22.50)	19 (10.75; 25)	0.046 *
Type 2 DM, *n* (%)	181 (88.73)	63 (85.14)	0.36
HbA1c (%)	7.50 (6.50; 8.50)	7.70 (7.70; 8.60)	0.981
Glucose (mg/dL)	138.8	143.8	0.752
Smoking, *n* (%)	35 (17.2%)	14 (18.9)	0.733
Alcoholism, *n* (%)	10 (4.9)	6 (8.1)	0.31
Comorbidities, *n* (%)			
Hypertension	143 (70.1)	56 (75.7)	0.362
Hypercholesterolemia	103 (50.5)	33 (44.6)	0.417
Retinopathy	55 (27)	35 (47.3)	0.001 *
CHD	47 (23)	19 (25.7)	0.648
PAD	83 (40.7)	42 (56.8)	0.020 *
Neuropathy	182 (89.2)	67 (90.5)	0.749
Wound duration, weeks	8 (3; 24.25)	12 (3.75; 26.50)	0.471
Healing time, weeks	12 (6; 21)	12 (6; 28)	0.576
History of foot ulcer, *n* (%)	86 (42.2)	37 (50)	0.275
History of amputation, *n* (%)	74 (36.3)	32 (43.2)	0.329
ABI	1.02 (0.69; 1.29)	1.07 (0.8; 1.4)	0.228
TcpO_2_ (mmHg)	35 (27; 42.75)	35 (25; 44)	0.804
Infection severity, *n* (%)			
Texas IIIB	121 (59.3)	32 (43.2)	0.020 *
Texas IIID	83 (40.7)	42 (56.8)	
Signs of local infection, *n* (%)			
Cellulitis	61 (29.9)	25 (33.8)	0.536
Purulent drainage	26 (12.7)	15 (20.3)	0.118
Blood creatinine level (mg/dL)	1.0 (0.86; 1.09)	1.52 (1.34; 2.28)	<0.001 *
eGFR (mL/min/1.73 m^3^)	70.1 (65.82; 77.92)	47.23 (38.1; 59.2)	<0.001 *

Note: Mean ± SD and median (inter-quartile range) for continuous variables. Percentage (%) for categorical variables. Abbreviations: HbA1c, glycosylated hemoglobin; CHD, coronary heart disease; PAC, peripheral arterial disease; ABI, ankle–brachial index; TcpO_2_, transcutaneous oxygen pressure; eGFR, estimated glomerular filtration rate. * *p* < 0.05.

**Table 2:** Frequency of foot ulcer locations.

Locations	Without DN (Group 1) (*n* = 204)	With DN (Group 2) (*n* = 74)
Forefoot, *n* (%)	195 (95.6)	71 (95.9)
Midfoot, *n* (%)	6 (2.9)	2 (2.7)
Hindfoot, *n* (%)	3 (1.5)	1 (1.4)

Abbreviation: DN, diabetic nephropathy.

**Table 3:** Frequency of microorganisms isolated from the DFUs.

Organisms	Without DN and Culture-Positive (Group 1) (*n* = 123)		With DN and Culture-Positive (Group 2) (*n* = 53)		*p* Value
	Number of Pathogens (*n*)	Percentage (%)	Number of Pathogens (*n*)	Percentage (%)	
Gram-positive bacteria (*n* = 122)			Gram-positive bacteria (*n* = 58)		
*Staphylococcus aureus* (MSSA)	32	26.23	16	27.58	0.569
*Staphylococcus aureus* (MRSA)	7	5.74	9	7.38	0.017 *
*Coagulase-negative Staphylococci* (CoNS)	45	36.89	16	27.59	0.413
*Streptococcus* spp.	12	9.84	6	10.35	0.753
*Enterococcus* spp.	13	10.66	4	6.90	0.534
*Corynebacterium* spp.	13	10.66	7	12.07	0.613
Gram-negative bacteria (*n* = 56)			Gram-negative bacteria (*n* = 21)		
*Escherichia coli*	6	10.71	6	28.57	0.120
*Pseudomonas aeruginosa*	18	32.14	5	23.81	0.348
*Klebsiella pneumoniae*	2	3.57	1	4.76	0.902
*Proteus* spp.	11	1.79	1	4.76	0.088
*Enterobacter* spp.	8	14.29	3	14.29	0.832
*Morganella morgani*	3	5.36	2	9.52	0.625
*Klebsiella oxytora*	3	5.36	0	0.00	0.251
*Serratia* spp.	1	1.79	1	4.76	0.538
*Citrobacter roseri*	1	1.79	0	0.00	0.510
*Providencia rettgeri*	1	1.79	0	0.00	0.510
*Acinetobacter* spp.	1	1.79	1	4.76	0.538
*Stenotrophomona maltophilia*	1	1.79	1	4.76	0.538

Abbreviations: DN, diabetic nephropathy; MSSA, methicillin-susceptible *Staphylococcus aureus*; MRSA, methicillin-resistant *Staphylococcus aureus.* * *p* < 0.05.

## Data Availability

Not applicable.
